# Single-cell RNA-seq reveals cell type-specific transcriptional signatures at the maternal–foetal interface during pregnancy

**DOI:** 10.1038/ncomms11414

**Published:** 2016-04-25

**Authors:** Andrew C. Nelson, Arne W. Mould, Elizabeth K. Bikoff, Elizabeth J. Robertson

**Affiliations:** 1 Sir William Dunn School of Pathology, University of Oxford, South Parks Road, Oxford OX1 3RE, UK

## Abstract

Growth and survival of the mammalian embryo within the uterine environment depends on the placenta, a highly complex vascularized organ comprised of both maternal and foetal tissues. Recent experiments demonstrate that the zinc finger transcriptional repressor *Prdm1*/Blimp1 is essential for specification of spiral artery trophoblast giant cells (SpA-TGCs) that invade and remodel maternal blood vessels. To learn more about functional contributions made by Blimp1+ cell lineages here we perform the first single-cell RNA-seq analysis of the placenta. Cell types of both foetal and maternal origin are profiled. Comparisons with microarray datasets from mutant placenta and *in vitro* differentiated trophoblast stem cells allow us to identify Blimp1-dependent transcripts enriched in SpA-TGCs. Our experiments provide new insights into the functionally distinct cell types present at the maternal–foetal interface and advance our knowledge of dynamic gene expression patterns controlling placental morphogenesis and vascular mimicry.

## Abstract (web summary)

The zinc finger transcriptional repressor *Prdm1*/Blimp1 is essential for remodelling maternal blood vessels in a subset of trophoblast cells. Here, the authors perform single-cell RNA-seq analysis on this Blimp1+ lineage, identifying functionally distinct cell types present at the maternal–foetal interface.

## Graphical abstract

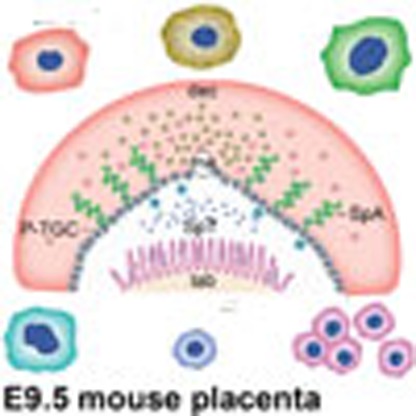

Placenta development is a highly dynamic process that requires coordinately regulated growth of the foetal capillary network in concert with expansion, and extensive remodelling of the maternal uterine vasculature by the invading embryonic trophoblasts. This specialized organ is essential for gas and nutrient exchange, production of hormones that regulate maternal and foetal physiology, and maternal tolerance of the foetal allograft.

Shortly after implantation the trophectoderm (TE) layer of the blastocyst expands and differentiates to form the extraembryonic ectoderm (ExE) and the ectoplacental cone (EPC), which in turn gives rise to the spongiotrophoblast (SpT) layer adjacent to maternal blood spaces. Subsequent placental morphogenesis leads to formation of a diverse set of trophoblast cell types distinguishable by localization, morphology and marker gene expression. A discrete trophoblast subset migrates into the maternal decidua to replace the lining of the spiral arteries and become spiral artery-associated trophoblast giant cells (SpA-TGCs). In addition, derivatives of the ExE-derived chorionic ectoderm give rise to subtypes that closely interact with foetal endothelial cells within the labyrinth region. Formation of these specialized trophoblast cell types is essential to insure adequate blood flow within the placenta during pregnancy. Defective remodelling of the maternal vasculature has been associated with preeclampsia, intrauterine growth restriction and miscarriage^1^,^2^.

The zinc finger transcriptional repressor *Prdm1/*Blimp1, originally identified as a silencer of β-interferon gene expression and a master regulator of plasma cell differentiation, controls cell fate decisions in the developing embryo and governs tissue homoeostasis in multiple cell types in the adult organism^3^. Developmental arrest of *Prdm1* mutant embryos at around embryonic day 10.5 (E10.5) is due to placental defects. *Prdm1* expression has been described in EPC-derived diploid trophoblasts and terminally differentiated giant cell types, including SpA-TGCs and glycogen trophoblasts (GlyTs), as well as endothelial cells within the labyrinth, and as yet ill-characterized maternal cells overlying the SpT^4^. Blimp1 function is required for specification of SpA-TGCs, proper expansion of the labyrinth region of the placenta and remodelling of the maternal vasculature^4^. Our microarray profiling of *Prdm1* mutant vs wild type E9.5 placenta revealed dramatically reduced expression of SpA-TGC-specific markers.

The principal confounding factor intrinsic to previous tissue-wide studies is the loss of cell type-specific expression data among the population average. In all likelihood, the signal-to-noise ratio in our experiments examining Blimp1-dependent transcripts in the placenta was substantially dampened by contributions from Blimp1-independent cell types. Recent advances in RNA-seq technology have made it possible to profile gene expression at a single-cell level. This technology is proving to be a particularly powerful tool for the analysis of complex tissues containing diverse cell populations. For example, elegant single-cell RNA-seq (scRNA-seq) experiments recently identified molecularly distinct cell types within the distal lung epithelium^5^.

Here we exploit scRNA-seq methodology to profile *Prdm1+* cell subpopulations in the developing placenta. Our data reveal differences between conventional foetal endothelial cells and so-called “vascular mimicry” functions performed by invading trophoblasts that remodel maternal spiral arteries. We describe transcriptional signatures characteristic of decidual stromal cells and uterine natural killer (uNK) cells present at the maternal–foetal interface, as well as trophoblast subsets responsible for hormone production during pregnancy. Collectively our data provides a blueprint for understanding transcriptional networks and signalling cues underlying trophoblast invasion and vascularity, and will be a valuable resource for future studies of mammalian placentation.

## Results

### Isolation of *Prdm1+* single cells from E9.5 placentae

**Figure 1:**
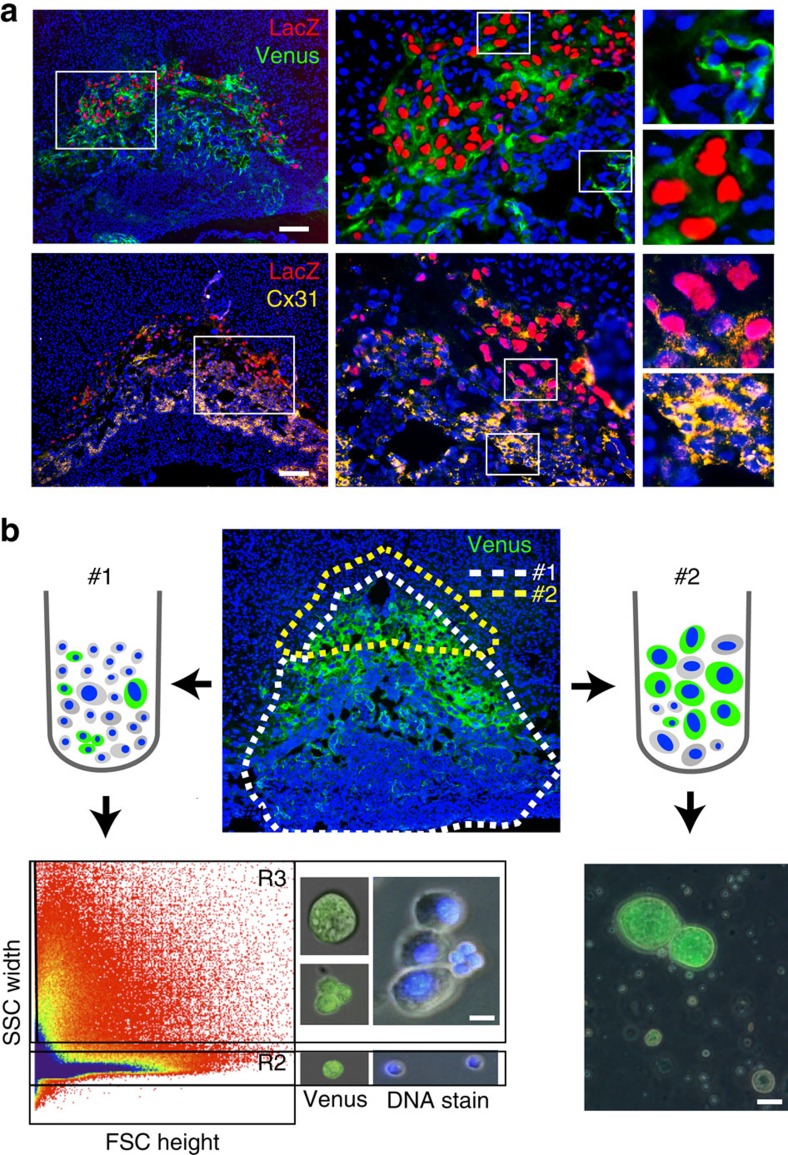
Strategies for isolation of single cells for scRNA-seq. (**a**) Expression patterns of paternally inherited *Prdm1*^*LacZ*^ knock-in and *Prdm1-Venus* transgenic reporter alleles at E9.5 substantially overlap (top). Cx31 is broadly expressed in diploid trophoblasts including LacZ+ diploid, but is undetectable in mature TGCs (bottom). Bar, 200 μm. (**b**) Cell isolation protocols for recovery of *Prdm1+* subpopulations. Bar, 10 μm.

We previously found that *Prdm1* is expressed in SpA-TGCs, GlyTs, a percentage of proliferative diploid trophoblasts within the SpT layer, foetal endothelial cells of the labyrinth, as well as undefined cell types of maternal origin within the decidua^4^. To characterize unique transcriptional signatures of diverse cell types in the developing placenta, we decided to profile Blimp1+ subpopulations by scRNA-seq. A *Prdm1-Venus* fluorescent BAC transgenic reporter previously used to study primordial germ cells *in vitro* and *in vivo*^6^,^7^ is strongly expressed in the placenta. Histological experiments confirmed co-expression of the paternally inherited *Prdm1-Venus* and our previously described LacZ knock-in reporter alleles^4^ in invasive TGCs, diploid trophoblasts, as well as a subset of endothelial cells in the labyrinth. In addition, ectopic *Venus* transgene expression was occasionally observed in LacZ negative cells (Fig. 1a).

Recent lineage tracing experiments revealed *Prdm1* expression in a subset of proliferative diploid trophoblasts representing SpA-TGC progenitors^4^. *Tfap2c* and *Gjb3* are known to be expressed in diploid trophoblasts and loss-of-function mutants display precocious trophoblast differentiation^8^,^9^,^10^. Immunostaining of *Prdm1*^*LacZ*^ placenta for the *Gjb3* gene product Cx31 revealed considerable overlap between LacZ and Cx31 expression in diploid *Prdm1+* trophoblasts within the SpT (Fig. 1a). Thus, *Tfap2c* and *Gjb3* are promising candidate marker genes for identification of SpA-TGC progenitors.

To isolate cells for scRNA-seq analysis, we carefully trimmed away maternal decidual tissue and enzymatically dissociated E9.5 placentae from embryos expressing the paternally derived Venus transgene. By flow cytometry we detected two distinct populations, namely R2 consisting of small single cells, and R3 consisting of cell clusters and large cells visible in forward/side scatter profiles (Fig. 1b). Venus+ cells, present in both regions were enriched by sorting (Method #1). Single diploid Venus+ cells were recovered within R2. In contrast, R3 contained Venus+ cells in small clusters. In addition, Hoechst 33342 staining identified TGCs containing large nuclei (Fig. 1b). However, recovery of Venus+ TGCs was disappointingly low, presumably due to their fragile nature.

To enrich for Venus+ TGCs we dissected the distal tip of the SpT layer together with the surrounding maternal tissue, followed by gentle enzymatic digestion (Fig. 1b). This alternative method (#2) yielded both large Venus+ cells, and Venus– maternal cells localized at the foetal–maternal interface (Fig. 1b). As summarized in Table 1, a total of 448 single cells or clusters of diploid cells were selected for further analysis. cDNAs (complementary DNAs) were initially screened for expression of *Prdm1*, *Venus*, *Prl7b1*, *Tfap2c* and *Gjb3* to distinguish maternal vs foetal populations, and identify putative SpA-TGCs and their progenitors. Using these selection criteria 78 samples including those positive for *Prdm1+*; *Venus+* (embryonic), *Prl7b1*, *Tfap2c* or *Gjb3*, as well as 18 of 39 *Prdm1+*; *Venus–* (maternal) samples were chosen for scRNA-seq analysis.

### Single-cell RNA-seq identifies discrete *Prdm1+* cell types

**Figure 2:**
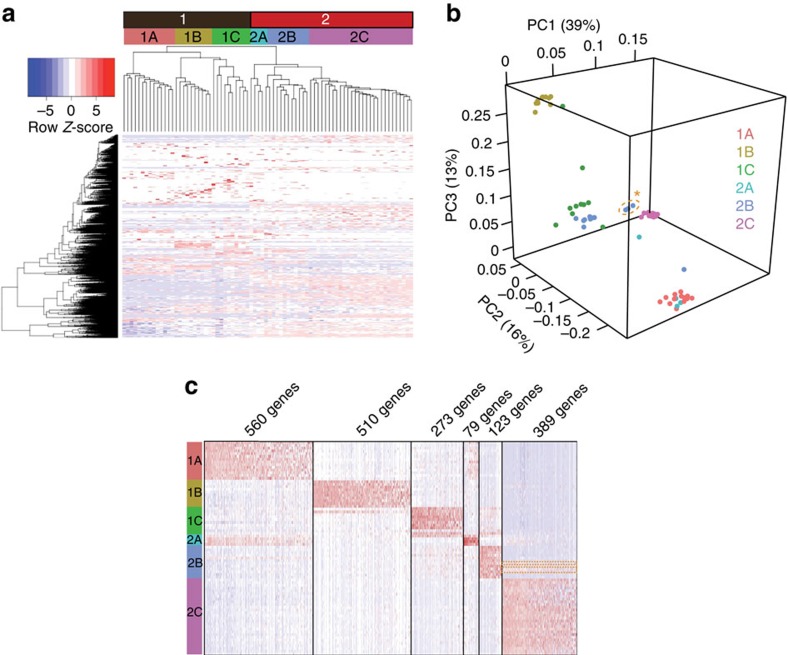
scRNA-seq analysis of *Prdm1+* placental cell populations demonstrate transcriptional profiles characteristic of distinct cell lineages. (**a**) Hierarchical clustering of scRNA-seq data from 78 samples identifies two groups (1 and 2) with three clear subdivisions, totalling six molecularly distinct populations (1A–C and 2A–C). Colour coded header bars indicate the given identifier for each group. (**b**) PCA of significantly variable genes on the basis of linear dimensional reduction revealing key relationships between groups. (**c**) Heatmap of genes significantly enriched within each individual group (*t*-test; *P*≤1 × 10^−3^). Group 2B cells expressing endothelial markers are outlined in orange.

Hierarchical clustering of the data revealed two distinct groups, each containing three clear subdivisions designated 1A–C and 2A–C (Fig. 2a). Principle component analysis (PCA) using all 15,402 detected genes revealed the same six distinct groups. Interestingly, group 2C contains numerous single cells together with the cell clusters recovered from R3, suggesting these correspond to the same cell type (Table 2). To further explore relationships between these six groups and identify genes that best describe the variance in the dataset, we performed linear dimensional reduction, followed by PCA on the resulting 1,007 genes (Fig. 2b). The top principle components reveal similarities shared between groups 1A and 2A, groups 1C and 2B, and a subset of group 2B with group 2C (Fig. 2b). In addition, we performed t-distributed stochastic neighbour embedding (tSNE) analysis using RaceID^11^. This methodology similarly identified the same six groups (Supplementary Fig. 1). Identification of genes significantly enriched in each group highlighted common features within these groups (Fig. 2c and Supplementary Data 1).

**Figure 3:**
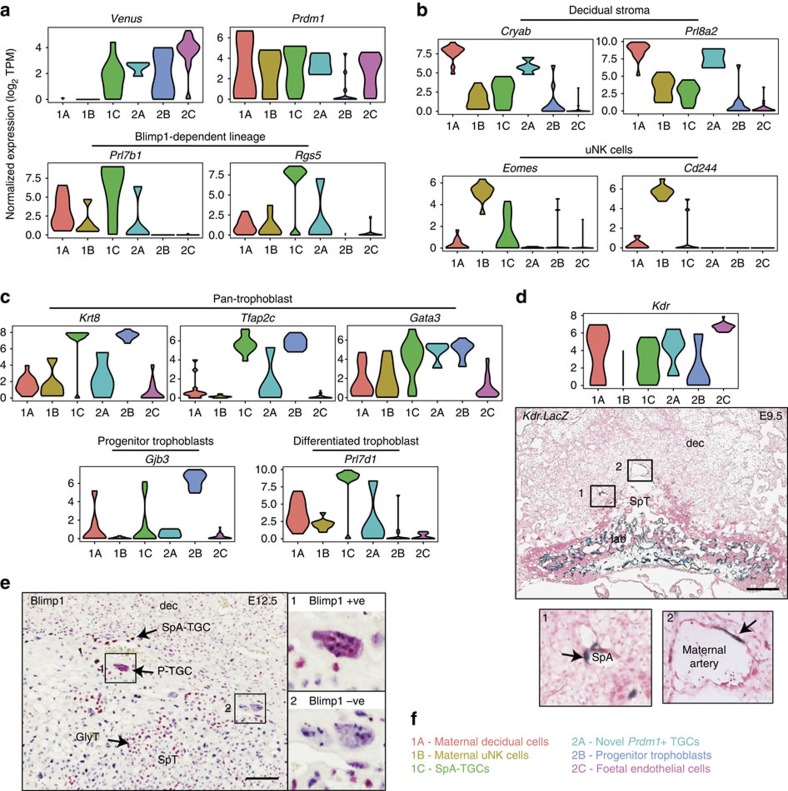
Marker genes defining discrete cell types. (**a**) Violin plots detailing expression levels of *Prdm1*, paternally inherited *Prdm1-Venus* reporter and *Prdm1*-dependent lineage markers *Prl7b1* and *Rgs5*. Scale is log_2_ tags per million (TPM). (**b**) Expression of decidual stroma markers *Cryab* and *Prl8a2* in group 1A; expression of uNK cell markers *Eomes* and *Cd244* revealing enriched expression in group 1B. (**c**) Violin plots of generic trophoblast markers *Krt8*, *Tfap2c* and *Gata3*, progenitor trophoblast marker *Gjb3* and *Prl7d1* marking differentiated trophoblasts. (**d**) Violin plot of *Kdr* expression, and X-gal staining of a paternally inherited *Kdr.LacZ* knock-in allele demonstrate expression in endothelial cells within the labyrinth and SpA-TGCs. Bar, 200 μm. (**e**) Anti-Blimp1 immunostaining of E12.5 placenta reveals Blimp1+ P-TGCs. Bar, 100 μm. (**f**) The cell types represented by the six groups. Abbreviations: dec, decidua; SpT, spongiotrophoblast; lab, labyrinth; SpA, spiral artery; TGC, trophoblast giant cell; SpA-TGC, spiral artery TGC; P-TGC, parietal TGC; GlyT, glycogen trophoblast.

Comparisons with well-characterized marker genes subsequently allowed us to identify individual cell types represented by these groups. Endogenous *Prdm1* transcripts were detectable in cells of all six groups (though only in a small minority of group 2B), (Fig. 3a). Paternally inherited *Prdm1-Venus* transcripts were selectively present in groups 1C–2C. Groups 1A and 1B are therefore of maternal origin. Group 1A expresses high levels of decidual stroma markers including *Prl8a2* (ref. 12) and *Cryab*^13^ (Fig. 3b). Analysis of the markers *Eomes* and *Cd244* (ref. 14) strongly suggests that group 1B corresponds to uNK cells (Fig. 3b). Consistent with this possibility, immunostaining experiments demonstrate that Eomes+ cells co-express the uNK cell marker *Dolichos biflorus* agglutinin lectin (DBA) (Supplementary Fig. 2a).

Venus+ group 1C cells of foetal origin express high levels of three generic trophoblast markers *Krt8*, *Tfap2c* and *Gata3*. In addition, group 1C expresses high levels of *Prl7d1*, reported to be widely expressed across diverse trophoblast subtypes^12^ (Fig. 3c). Interestingly expression of *Gjb3* and *Prl7d1* seems to be mutually exclusive, suggesting that *Gjb3* expression may be restricted to an immature subpopulation, whereas *Prl7d1* is a marker of differentiating trophoblasts.

Group 1C cells were also found to be strongly express Blimp1-dependent markers *Prl7b1* and *Rgs5* (Fig. 3a)^4^ suggesting these cells represent either invasive SpA-TGCs or GlyTs. To distinguish SpA-TGCs vs GlyTs, we analysed several additional members of the rodent Prolactin (Prl) gene family, known to be expressed by distinct trophoblast subtypes^12^. Group 1C cells express high levels of *Prl2c2*, *Prl2c3*, *Prl2c5* and *Prl5a1*, known to be strongly expressed in SpA-TGCs, but not GlyTs (Supplementary Fig. 3a). In addition, group 1C cells express the vascular signalling receptor *Kdr* (formerly *Flk1*/*Vegfr2*; Fig. 3d)^15^. Interestingly X-gal staining experiments demonstrate expression of a paternally inherited *Kdr.LacZ* reporter^15^ in SpA-TGCs as well as foetal endothelial cells, confirming that group 1C contains SpA-TGCs (Fig. 3d).

We found that group 2A *Venus+*; *Prdm1+* cells weakly co-express *Krt8* and *Prl7d1* (Fig. 3a,c). Downregulated *Krt8* expression has been linked to endoreduplication in parietal TGCs (P-TGCs)^16^. Consistent with their endoreduplicating cell status, group 2A cells express high levels of multiple histones, (Supplementary Fig. 3b). *Prdm1+* P-TGCs were not previously observed at E9.5 (ref. 4). However, we found here that *Prdm1+* P-TGCs were detectable at slightly later stages, in E12.5 placenta (Fig. 3e). It is therefore tempting to speculate that these moderately sized group 2A cells, largely devoid of *Prl* gene expression correspond to immature *Prdm1+* P-TGCs. Group 2B diploid cells strongly express trophoblast markers including *Gjb3*, and as discussed below appear to represent TGC progenitors. The group 2C *Venus+* cells, expressing high levels of *Kdr*, but low levels of trophoblast markers, and in all likelihood correspond to *Prdm1+* allantoic endothelial cells (Fig. 3d).

Intriguingly, numerous transcripts were expressed by both group 2A (large cells of foetal origin) and 1A (decidual stromal cells), including the decidual marker genes *Prl8a2* and *Cryab* (Figs 2c and 3b). To exclude technical issues such as the presence of doublets or cross-contamination during single-cell isolation, we expanded the analysis to identify additional transcripts. The results shown in Supplementary Fig. 4 clearly demonstrate that many of the group 1A highly expressed genes are entirely absent from group 2A. While it is formally possible that these surprising results could potentially reflect fusion of a subpopulation of foetal TGCs with maternal decidual cells, we suggest that the more plausible explanation is that these curious findings reflect *bona fide* transcriptional signatures of the discrete group 1A and group 2A cells both influenced by common signalling events at the maternal–foetal interface. It will be interesting to compare 1A and 2A transcriptional signatures as documented in the present report with additional pregnancy single-cell RNA-seq datasets as they become available.

### Evidence that group 2B are trophoblast progenitors

**Figure 4:**
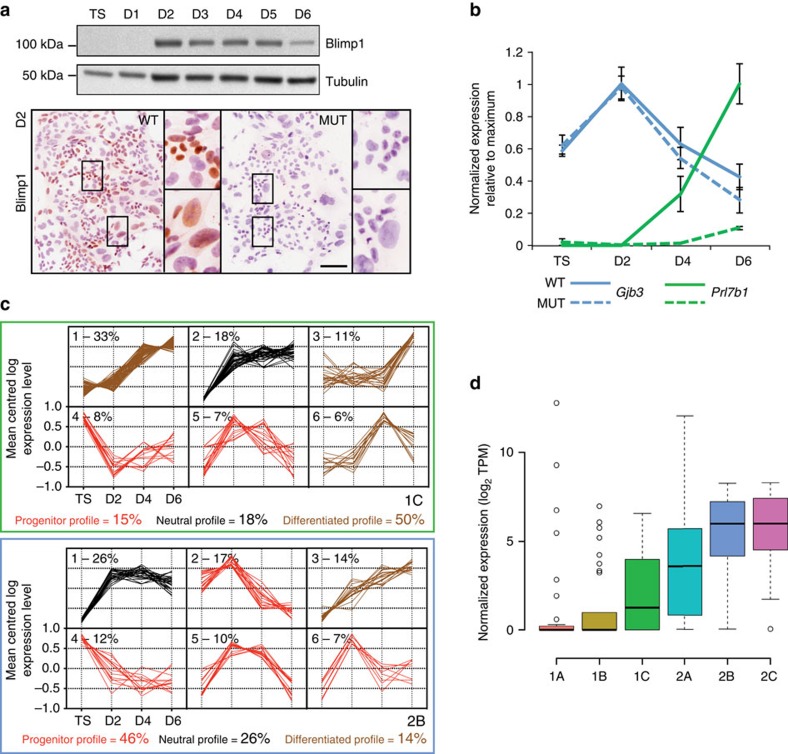
scRNA-seq data together with transcriptional profile changes during *in vitro* TS cell differentiation implicates *Gjb3+* group 2B cells as trophoblast progenitors. (**a**) The appearance of *Prdm1*/Blimp1+ diploid cells beginning at around day 2 during *in vitro* differentiation. Bar, 100 μm. (**b**) *Gjb3* is transiently upregulated early in TS cell differentiation, whilst the SpA-TGC marker *Prl7b1* is induced at late stages during wild type but not *Prdm1* mutant differentiation. Data represents mean±SEM of 6–10 samples per genotype at each stage (**c**) k-means clustering of genes defining SpA-TGCs (1C) and progenitor trophoblasts (2B). The six largest clusters per sub-group. Clusters are categorized as displaying a progenitor profile—containing genes either highest in TS cells or transiently upregulated in early differentiation; a differentiated profile—genes highest in later differentiation; or a neutral profile—genes upregulated early on exit from the TS state and remaining high throughout differentiation. Percentages of genes corresponding to progenitor, neutral or differentiated profiles are indicated. (**d**) scRNA-seq median expression per group of cell cycle-regulated genes. Boxed region, bar and whiskers represent interquartile range, median and 95% central range of the data. Outliers indicated by open circles.

Self-renewing, multipotent trophoblast stem (TS) cells derived from either blastocysts or the ExE differentiate *in vitro* to form mature trophoblast cell types^17^. To further characterize about the cell types defined by our scRNA-seq dataset and learn more about Blimp1-dependent transcripts we isolated a panel of wild type and *Prdm1*-deficient TS cell lines. Western blot and immunostaining experiments demonstrate *Prdm1*/Blimp1 expression is upregulated by day 2 of differentiation in diploid trophoblasts and maintained in a subset of TGCs that undergo endoreduplication (Fig. 4a and Supplementary Fig. 5a). *Gjb3* expression is readily detectable in wild type control TS cells whereas the SpA-TGC marker *Prl7b1* appears later as *Gjb3* declines (Fig. 4b).

To test the hypothesis that *Gjb3+* diploid cells are progenitor trophoblasts, while large *Prl7b1+* cells are terminally differentiated, TGCs we performed a microarray timecourse and then analysed the expression characteristics of genes enriched in groups 2B and 1C. Group 2B cells display a clear bias towards genes showing highest expression in TS cells or early differentiation, consistent with the idea that these are trophoblast progenitors (Fig. 4c). To further explore this possibility we assessed expression of cell cycle markers^18^ represented in our scRNA-seq dataset. Both foetal endothelial cells (2C) and *Gjb3+* trophoblasts (2B) showed the highest expression of cell cycle genes (Fig. 4d). Collectively the results demonstrate that group 2B cells bear all the expected hallmarks of trophoblast progenitors.

**Figure 5:**
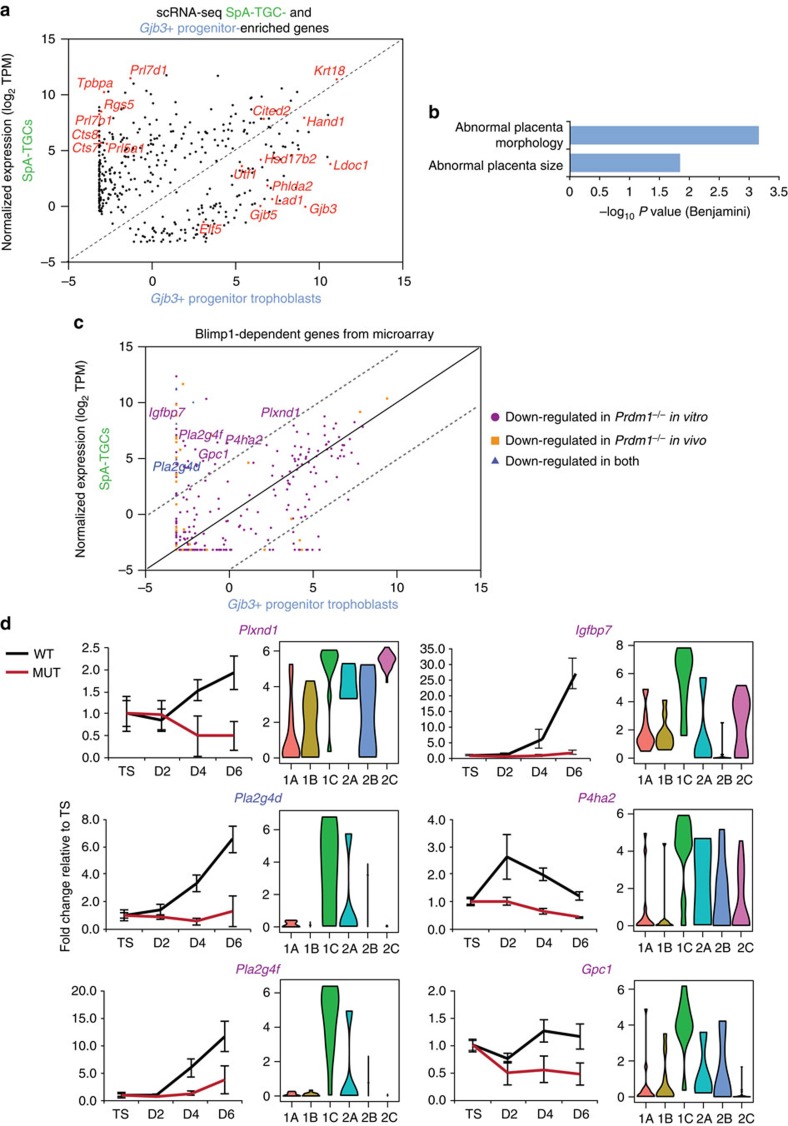
Genes expressed by *Gjb3+* progenitor trophoblasts are required for normal placenta development, whilst Blimp1-dependent genes expressed by SpA-TGCs regulate vascular function. (**a**) Scatter plot of the 396 genes enriched in SpA-TGCs and *Gjb3+* progenitors presented in Figure 2c. A subset of SpA-TGC markers and genes expressed in progenitors with deleterious placental phenotypes are indicated. Plotted are mean gene expression values within each group. (**b**) Functional annotation analysis indicating MGI phenotypes associated with genes enriched in *Gjb3+* progenitors. (**c**) Scatter plot of genes downregulated in *Prdm1* mutants *in vivo* or *in vitro*. Plotted are median gene expression values within each group. (**d**) Relative expression of genes downregulated in mutant and wild type TS cells during *in vitro* differentiation (left) in comparison with scRNA-seq data (right). Data represents mean±s.e.m. of 6–10 samples per genotype at each stage.

To distinguish SpA-TGCs from *Gjb3+* trophoblast progenitors we compared genes enriched in groups 1C and 2B. We found that markers strongly expressed by invasive trophoblasts, *Cts7*, *Cts8* (ref. 19) and *Tpbpa*^20^ are absent in *Gjb3+* trophoblasts (Fig. 5a). Interestingly, *Gjb3+* trophoblasts display enriched expression of genes with deleterious placental loss-of-function phenotypes (Fig. 5b).

### Identification of SpA-TGC-enriched Blimp1-dependent genes

Next we examined our microarray data to search for Blimp1-dependent transcripts. The profiles of wild type and *Prdm1* mutant TS cells were initially indistinguishable. However, significant divergence was observed subsequently in differentiating cultures (Supplementary Fig. 5b). As expected Blimp1-deficient TGCs fail to express the SpA-TGC marker *Prl7b1* (Fig. 4b). Functional analysis of transcripts downregulated in *Prdm1*^*–/–*^ trophoblasts showed strong enrichment for genes required for blood vessel and extraembryonic tissue development, as well as normal embryonic growth (Supplementary Fig. 5c). Moreover, comparison of the present microarray dataset with results of our previous profiling experiments analysing wild type vs mutant E9.5 placenta^4^ revealed a small subset of overlapping downregulated transcripts including several SpA-TGC marker genes, that is, *Prl7b1* and *Rgs5* (Supplementary Fig. 5d).

We exploited our scRNA-seq dataset to directly compare Blimp1-dependent genes expressed by SpA-TGCs or *Gjb3+* progenitor trophoblasts (Fig. 5c). As shown in Fig. 5d numerous Blimp1-dependent transcripts detectable in differentiating TS cell cultures, including *Plxnd1* and *Pla2g4d* show SpA-TGC-enriched expression *in vivo*. Thus their absence in *Prdm1* mutants probably reflects selective loss of the SpA-TGC lineage.

### Functionality predicted by cell type-specific transcription

The six cell types defined by our scRNA-seq analysis and their corresponding marker gene expression fits well with their defined size differences, that is, large TGCs vs relatively small endothelial cells (Table 2). Consistent with the identification of group 1C as SpA-TGCs and group 2B as immature trophoblast progenitors both these cell populations show strikingly similar expression profiles (Fig. 2b). Interestingly, 3 of the 12 group 2B cells express genes characteristic of group 2C endothelial cells (orange box in Fig. 2c), highlighting the functional similarities between trophoblasts and endothelial cells^21^. Notably, the one exceptional small cell within group 1C expressing high levels of *Gjb3* together with low levels of *Prl7b1* and *Prl7d1*, potentially represents a SpA-TGC progenitor undergoing terminal differentiation (Fig. 3a,c).

**Figure 6:**
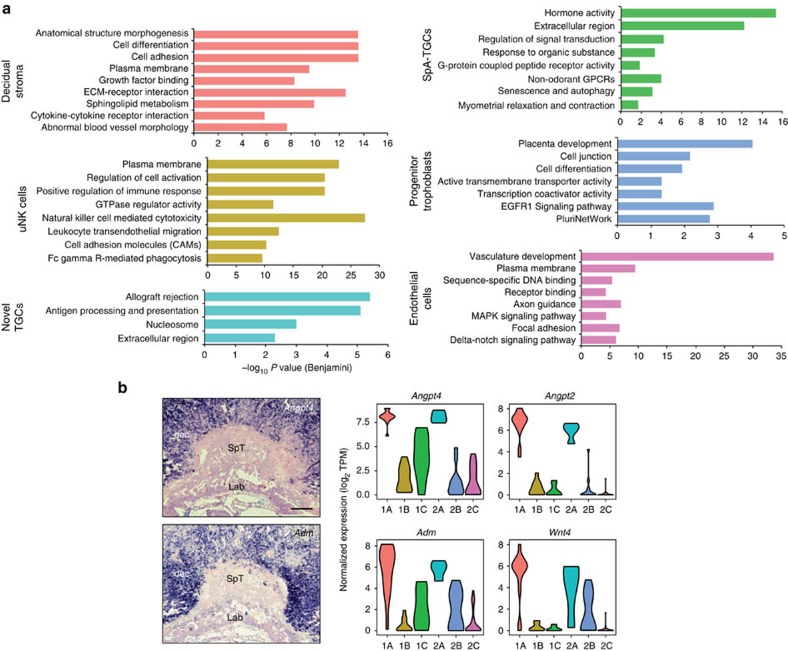
Functional annotation analysis reveals discrete activities of each cell type. (**a**) Functional annotation of genes corresponding to each of the groups as indicated in Figure 2c. the selected Gene Ontology, KEGG Pathway, Wikipathway and MGI phenotype terms are shown. Benjamini and Hochberg corrected –log_10_ *P* value from hypergeometric test is reported. (**b**) Decidual stromal cells express high levels of secreted factors regulating vascular fates. Bar, 200 μm.

**Figure 7:**
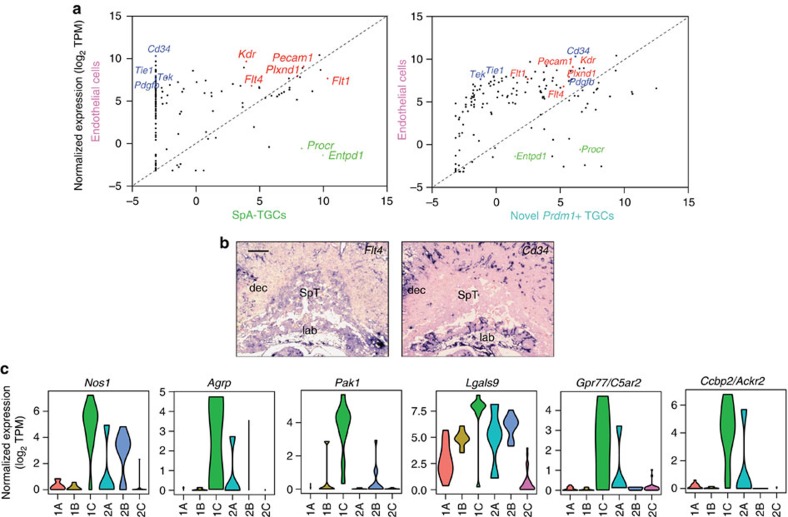
The transcriptional signatures of SpA-TGCs and endothelial cells only partially overlap. (**a**) Scatter plot of endothelial markers identified by Takase *et al*. demonstrates that the majority are expressed in allantoic endothelial cells but are not detected in SpA-TGCs. Scatter plot of endothelial cell genes compared to novel *Prdm1+* TGCs (group 2A) reveals detectable expression of almost all endothelial markers in novel *Prdm1+* TGCs. Plotted are median gene expression values within each group. (**b**) ISH reveals widespread expression of the endothelial marker *Flt4* throughout trophoblasts, but more restricted *Cd34* expression. Bar, 200 μm. (**c**) Genes enriched in SpA-TGCs are involved in immune tolerance, hormone signalling, vasodilation and trophoblast invasion.

Functional annotation analysis of the genes differentially expressed by each of the six groups provides insight into their distinct functional activities. For example, the decidual stromal cell transcriptome governs placental morphogenesis, differentiation, blood vessel morphology and regulation of endothelial behaviour through sphingolipid metabolism. Interestingly, we found the peptide hormone *Adm* is highly expressed in this cell population (Fig. 6b). *Adm*, previously identified as a P-TGC marker is also a key regulator of uNK cells^22^. uNK cells are thought to destroy the surrounding vascular smooth muscle cells and restrict the extent of SpA-TGC invasion during spiral artery remodelling^23^,^24^. The present results strongly suggest that decidual stromal cells, also regulate *Adm*-mediated recruitment of uNK cells. Group 2A cells express *Adm* and the P-TGC marker *Cyp11a1* (ref. 25) (Supplementary Fig. 3c). Mature P-TGCs were recently shown to express the cell–cell adhesion molecule *Cd34* (ref. 26). Interestingly group 2A cells similarly express *Cd34*, consistent with the possibility they represent immature P-TGCs (Fig. 7a).

**Figure 8:**
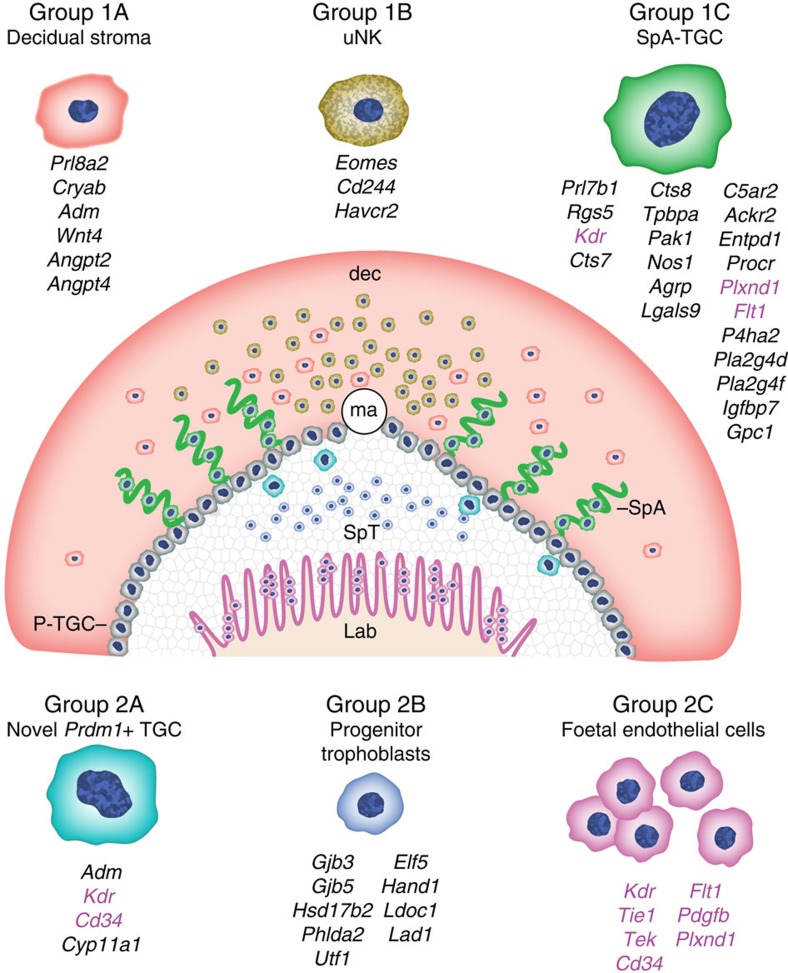
Summary of key marker genes identifying each cell type. Overview summary of the locations of the profiled cell types within E9.5 placenta and key identified markers discussed above are indicated. Vascular markers are in pink. Abbreviations: dec, decidua; ma, maternal artery; SpA, spiral artery; SpT, spongiotrophoblast layer; lab, labyrinth; P-TGC, parietal trophoblast giant cell.

The cell type-specific transcriptional signatures described here refine our understanding of the pathways controlling placental vascularity. For example, *Wnt4* (ref. 27) and the Angiopoietins *Angpt2* (ref. 28) and *Angpt4* (ref. 29) were found to be highly expressed in the decidual stroma (Fig. 6b), whereas the Angiopoietin receptors *Tek* and *Tie1*, as well as *Cd34* and pericyte recruitment factor *Pdgfb*^30^, largely restricted to endothelial cells were undetectable in SpA-TGCs (Fig. 7a). On the other hand, numerous other endothelial cell markers were expressed at similar levels in both SpA-TGCs and endothelial cells, for example, *Flt4* and *Pecam1* (Fig. 7a,b and Supplementary Fig. 3d). Overall our experiments have identified a plethora of novel genes regulating placental development and trophoblast function. Figure 8 presents a summary overview of the cell type-specific transcriptional signatures defined in the present report.

## Discussion

Gene targeting studies have revealed essential functions performed by key transcription factors, signalling molecules and membrane glycoproteins controlling cell–cell interactions during placental development^31^. Changes in gene expression profiles associated with many of these loss-of-function phenotypes have also been characterized via microarray experiments. Our recent experiments demonstrate that developmental arrest of *Prdm1* mutant embryos at around E10.5 is associated with loss of the invasive SpA-TGC cell lineage^4^. In addition, lineage tracing experiments identified a population of proliferating Blimp1+ diploid cells present within the SpT that give rise to mature SpA-TGCs, canal TGCs and GlyTs.

The SpA-TGC lineage mediates key functions during pregnancy including decidual invasion, vascular mimicry leading to increased blood flow, evasion of maternal immune response, and hormonal regulation of maternal physiology and behaviour. However, isolation and characterization of this crucially important mature TGC subset has been technically challenging due to their fragile nature and intimate association with the maternal vasculature. Consequently, the transcriptional programmes governing their complex behaviour remain ill defined. The present scRNA-seq analysis provides new insights into their cell type-specific transcriptional signatures and defines the key functionally distinct cell subpopulations present at the maternal–foetal interface, as summarized in Fig. 8.

We identified a number of transcripts selectively expressed by SpA-TGCs, including genes consistent with their invasion, vascular and endocrine functions (Figs 5c,d and 7c). *Pak1* is known to promote trophoblast invasion in gestational trophoblastic disease in human^32^. Nitric oxide production via *Nos1* promotes vasodilation and increased blood flow. The peptide hormone *Agrp* is linked to obesity and enhanced appetite^33^. The semaphorin receptor *Plxnd1* expressed in both endothelial cells and SpA-TGCs, regulates Vegf signalling^34^ by promoting expression of soluble *Flt1* (sFlt1), a Vegf decoy receptor, to limit angiogenic potential^35^. sFlt1 is known to be produced by SpA-TGCs^36^. Two Blimp1-dependent phospholipase A2 family enzymes, *Pla2g4d* and *Pla2g4f*, potentially act downstream Vegf receptor *Kdr* to promote production of the vasodilator prostacyclin^37^. The hypoxia-induced extracellular matrix remodelling factor *P4ha2* (ref. 38), marker of tumour vasculature *Igfbp7* (ref. 39), as well as *Gpc1*, a co-receptor for heparin-binding growth factors such as FGFs and modulator of angiogenesis and metastasis of human and mouse tumours^40^ were also downregulated in the absence of Blimp1. Interestingly, the present experiments demonstrate for the first time that SpA-TGCs express the Vegf receptor *Kdr*. Recent studies have shown that Vegf activates Blimp1 expression in tumour vasculature^41^. It will be interesting to learn whether Vegf signalling similarly upregulates Blimp1 expression during SpA-TGC specification.

Our results clearly demonstrate that SpA-TGCs are well equipped to suppress harmful consequences of vascular remodelling. The galectin *Lgals9* attenuates immune response by signalling via its receptor Havcr2 on immune cells (Supplementary Fig. 3e)^42^. In addition, *Gpr77/C5ar2* proposed to be a decoy receptor involved in evasion of the complement pathway^43^, as well as *Ccbp2/Ackr2*, a decoy receptor thought to prevent spontaneous miscarriage by attenuating inflammatory responses^44^, both G-protein coupled receptor family genes, were found to be specifically expressed by SpA-TGCs (Figs 6a and 7c). SpA-TGCs strongly express both *Entpd1* and *Procr* known to prevent thrombosis and inflammation (Fig. 5a)^45^,^46^. *Procr* loss-of-function mutants exhibit mid-gestation lethality due to placental thrombosis.

Comparisons of uNK cells profiled here with splenic NK1.1+ RNA-seq datasets strengthens the idea that uNK cells represent a unique NK subset. NK marker genes including *Eomes, Cd122, Cd123* and *Prf1 (perforin)*^14^ were strongly expressed, but group 1B (uNK) cells lack expression of numerous other genes associated with NK activation (Supplementary Fig. 2b). *Ifnγ* is known to be a Blimp1 target in T effector cells^3^ and human NK cells^47^. Consistent with repressed cytolytic function, here we found Blimp1+ uNK cells express low levels of *Ifnγ* compared with splenic NK cells. It will be interesting to further investigate NK cell heterogeneity using scRNA-seq technology.

Recent advances in microfluidics and automation technologies have proven invaluable for profiling single cells with similar sizes and morphological characteristics in studies where prior enrichment for rare cell types and maintenance of viability were not complicating factors^48^,^49^. However, diverse cell subsets within the placenta display vastly different sizes and adhesion properties. Here TGCs exhibiting a broad range of morphologically distinct features were manually isolated. The present experiments provide the first comprehensive survey of gene expression profiles in individual placental cell types. Our data establishes hierarchical relationships between these cell type-specific transcriptional profiles, and reveals numerous novel marker genes for future study of placental development. Building on previous placental disruption methods^50^,^51^, here we established reliable methods suitable for isolating cell types of interest, subsequently selected by brute force screening of libraries for marker genes. The present study provides new insights into key aspects of mammalian placental development, while informing future experimentation into vascular mimicry, immune tolerance and hormone signalling.

## Methods

### Animals

The *Prdm1*^*BEH*^ null allele, *Prdm1*^*Cre-nLacZ*^, *Prdm1-Venus* and *Kdr*^*LacZ*^ mouse strains were genotyped according to published protocols^4^,^6^,^15^,^52^. Males from the various reporter strains (C57BL/6J, 2–4 months of age) were crossed with wild type females (CD-1 from Charles River Laboratories, 6–12 weeks of age) to ensure reporter gene expression marked only tissues of embryonic origin. Experiments were performed in accordance with Home Office regulations.

### TS cell culture and differentiation

TS cell lines were derived from individual blastocysts obtained from *Prdm1*^*+/BEH*^x *Prdm1*^*+/BEH*^intercrosses and genotyped as previously described^17^,^52^. TS cells were adapted for growth under defined conditions^53^. To induce differentiation trypsinized TS cells were plated in the absence of FGF4, TGFβ1 and heparin sulphate. Four independent cell lines of each genotype were used for all experiments.

### Histology

For immunohistochemistry tissue was fixed in 4% paraformaldehyde (PFA) in PBS overnight at 4 °C, paraffin embedded and sectioned (6 μm). For immunofluorescence tissue was fixed overnight in 1% PFA in PBS at 4 °C, cryo-protected in 30% sucrose, embedded in O.C.T. Compound (Tissue-Tek) and sectioned (10 μm). In some instances X-gal staining was performed on cryosections followed by immunostaining. For immunostaining, sections were blocked for 1 h in 5% foetal bovine serum/0.2% bovine serum albumin in PBS at room temperature followed by overnight incubation with primary antibody in blocking buffer at 4 °C. For immunostaining of cells grown on Matrigel-coated glass coverslips, samples were fixed in 4% PFA in PBS for 10 min at room temperature, permeabilised with 2.5% Triton-X100 in PBS for 10 min, blocked with 10% Donkey serum for 1 h followed by overnight incubation with primary antibody in blocking buffer at 4 °C. Primary antibodies were detected by incubation with an appropriate secondary, and in some cases a tertiary Alexa Fluor-conjugated antibody (Molecular probes). Sections were mounted in VECTASHIELD containing DAPI (Vector Laboratories). Primary antibodies used were rabbit anti-GFP (Invitrogen, A-31851, 1:500), rabbit anti-Cx31 (Alpha Diagnostics, CX31-A, 1:200), monoclonal rat anti-Blimp1 (clone 5E7, Santa Cruz SC-130917, 1:500) and rabbit anti-Eomes (Abcam, ab23345, 1:100).

For *in situ* hybridization tissue was fixed overnight in 4% PFA, dehydrated in ethanol, paraffin embedded, and sectioned (6 μm). Riboprobe synthesis and RNA *in situ* hybridization was performed according to standard protocols using mid-plane sections. For *Adm* and *Angpt4* PCR fragments amplified from E9.5 placenta were cloned into pBlueScript KS- using primers indicated in Supplementary Table 1 prior to probe synthesis. IMAGE clone 3966337 was used for *Cd34*. The *Flt4* probe was a kind gift from Heiko Lickert.

### Isolation of single cells and cell clusters

Placentae from CD-1 females mated to *Prdm1-Venus* males were harvested at E9.5 and the yolk sac and decidual tissue trimmed away being careful to leave the labyrinth and SpT layers intact. *Prdm1-Ven*us*+* placentae were identified by fluorescent imaging of the corresponding embryo.

Pools of three placentae were finely minced with spring scissors and digested in Liberase TM (Roche, 0.325 Wunsch u ml^−1^) and DNase 1 (Sigma-Aldrich, 20 Kunitz u ml^−1^) in DMEM supplemented with 15% FCS, 10% Medium NCTC-109 (Gibco), 15 mM HEPES, 0.1 mM non-essential amino acids, 2 mM Glutamine and 1% Hybri-Max OPI Media Supplement (Sigma-Aldrich). Digestion was performed for 45 min at 37 °C in a shaking incubator. Samples were vigorously pipetted every 10 min to assist tissue disassociation.

After centrifugation, cell pellets were resuspended, incubated in Accumax (Sigma-Aldrich) for 10 min at 37 °C then filtered through 100 μm mesh. Venus+ single diploid cells, diploid cell clusters and giant cells were gated on forward vs side scatter profiles and GFP fluorescence using a MoFlo XDP Flow cytometer (Beckman Coulter). Viable cells were identified by 7AAD exclusion. High-resolution images were captured using an ImageStreamX Mk II imaging flow cytometer (AMNIS) and analysed using IDEAS software (AMNIS).

Due to low recovery of Venus+ giant cells using FACS, giant cells were also manually picked from SpT tissue after enzymatic disassociation. To enrich for Venus+ TGCs, the distal tip of the SpT layer proximal to the maternal artery was used.

### Preparation of single-cell cDNAs and sequencing

Cell suspensions were diluted to low density and single cells or clusters of 2–3 cells specifically selected under phase-contrast microscopy and picked directly into lysis buffer by mouth pipette, followed by synthesis and amplification of cDNA precisely as previously described^54^. To identify potential cells of interest, qPCR was performed using QuantiTect SYBR Green PCR kits on a Rotor-Gene Q (QIAGEN) using primer pairs detailed in Supplementary Table 1. Purified cDNAs from cells of interest were then subjected to Illumina sequence library preparation and sequencing on an Illumina HiSeq 2500.

### RNA-seq data analysis

Sequence reads were mapped to the mm9 mouse genome release with Stampy using default parameters^55^. Read pairs with identical outer coordinates were reduced to a single pair. Numbers of remaining properly paired reads per sample are reported in Supplementary Table 2. Reads were then assigned to gene annotations of Ensembl release 67 using HTSeq-count^56^. To normalize raw gene intensities between libraries, scaling factors were calculated using a trimmed mean of *M*-values between each pair of samples within the six identified groups using edgeR^57^. Hierarchical clustering was performed in R. Further analysis was performed using Seurat, an R package for single-cell genomics, which has been extensively described previously^49^,^58^. We filtered out cells expressing <3,000 genes, and genes detected in <3 cells. This resulted in 78 cells expressing a total of 15,402 genes. Details of number of genes detected per cell per group are provided in Supplementary Fig. 6. Dimensional reduction was performed and significant principle components (*P*<1 × 10^−2^) identified on the basis of 200 random samplings, each time permuting 1% of genes. PCA projection was then performed and the top 300 significant genes (*P*≤1 × 10^−5^) from each significant principle component selected giving a total of 1,007 genes describing the variance in the dataset. Significant genes identified by this method were used for PCA using princomp in R. Genes significantly enriched in each group revealed by hierarchical clustering were identified using the t-test algorithm in Seurat (*P*≤1 × 10^−3^ was considered significant). Identification of cell groups using RaceID and *t*-distributed stochastic neighbour embedding was performed using the spearman clustering metric^11^. Functional annotation of the resulting gene lists relative to KEGG Pathways, Wikipathways, mammalian phenotype and Gene Ontology terms was performed with WebGestalt using default parameters^59^. Endothelial markers referred to in Fig. 7a,b were defined by Takase *et al*.^60^.

### Western blot analysis

Cell lysates were analysed by western blot, using a rat anti-Blimp1 monoclonal antibody (Santa Cruz, sc-130917, 1:500) and rabbit anti-Tubulin polyclonal antibody (Santa Cruz, sc-9104, 1:1,000). All uncropped western blots can be found in the Supplementary Fig. 7.

### Microarray experiments

TS and TG cells were directly lysed on plates at the indicated timepoints and total RNA prepared with an RNeasy MiniKit (Qiagen) with DNase treatment according manufacturers protocol. Microarray experiments were performed using Illumina Mouse WG-6 v2 Expression BeadChips. Microarray data was analysed using rank-invariant normalization and Mann–Whitney options of the Gene Error Model Expression Analysis Module V1.6.0 of GenomeStudio V2009 (Illumina). K-means clustering was performed using Cluster 3.0 (ref 61). Phenotypic analysis of differentially expressed genes was performed using Enrichr^62^.

## Additional information

**How to cite this article:** Nelson, A. C. *et al*. Single-cell RNA-seq reveals cell type-specific transcriptional signatures at the maternal–foetal interface during pregnancy. *Nat. Commun.* 7:11414 doi: 10.1038/ncomms11414 (2016).

## Supplementary Material

Supplementary Information Supplementary Figures 1-7 and Supplementary Tables 1-2

Supplementary Data 1 Lists of genes significantly upregulated in each group identified by scRNA-seq and associated P values

## Figures and Tables

**Table 1:** Summary of samples selected for scRNA-seq.

**Method**	**Small cells**	**Clusters**	**Large cells**	**Total**
#1	116	24	116	256
#2	0	0	192	192
Total	116	24	308	448

**Table 2:** Details of sample type in each group.

**Group**	**Small cell**	**Cluster**	**Large cell**	**Total**
1A	0	0	14	14
1B	0	0	10	10
1C	1	0	9	10
2A	0	0	4	4
2B	12	0	0	12
2C	11	17	0	28
Total	24	17	37	78
