# Publisher Correction: Single-cell RNA-seq reveals cell type-specific transcriptional signatures at the maternal–foetal interface during pregnancy

**DOI:** 10.1038/ncomms16219

**Published:** 2018-11-23

**Authors:** Andrew C. Nelson, Arne W. Mould, Elizabeth K. Bikoff, Elizabeth J. Robertson

Nature Communications 7: Article number: 11414; DOI: 10.1038/ncomms11414 (2016), Published 04 25 2016; Updated 11 23 2018

In the original version of this Article, the accession codes section within the Methods was inadvertently omitted. This section has now been added in both the PDF and HTML versions of the Article.

