# Supplementary material for: Single-cell RNA-seq reveals cell type-specific transcriptional signatures at the maternal–foetal interface during pregnancy
Source: Nat Commun. 2016 Apr 25;7:11414. doi: 10.1038/ncomms11414 (PMC4848515; doi:10.1038/ncomms11414)
Supplement: Supplementary Information — Supplementary Figures 1-7 and Supplementary Tables 1-2 [file ncomms11414-s1.pdf]

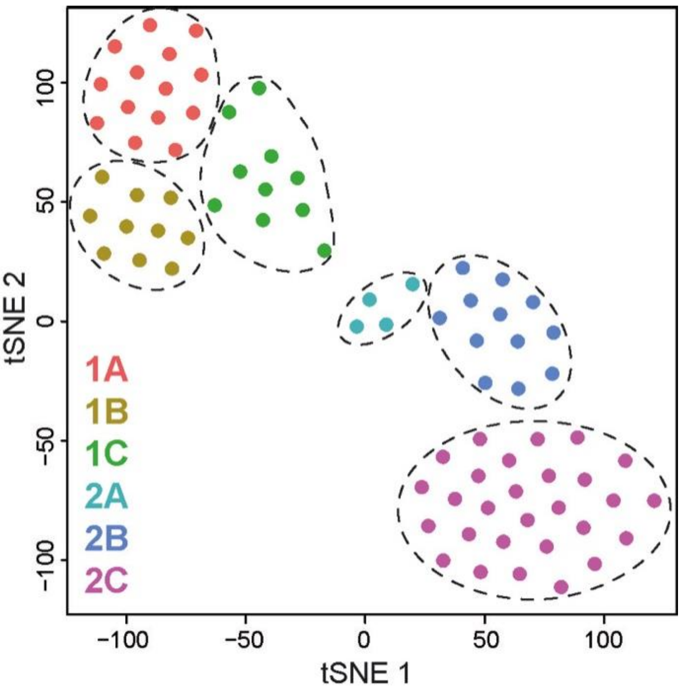

Supplementary Figure 1. tSNE plot indicating cell classifications generated using RaceID. 1A – Decidual stromal cells; 1B – uterine NK cells; 1C – SpA-TGCs; 2A – Novel *Prdm1+* TGCs; 2B – Progenitor trophoblasts; 2C – Foetal endothelial cells.

a

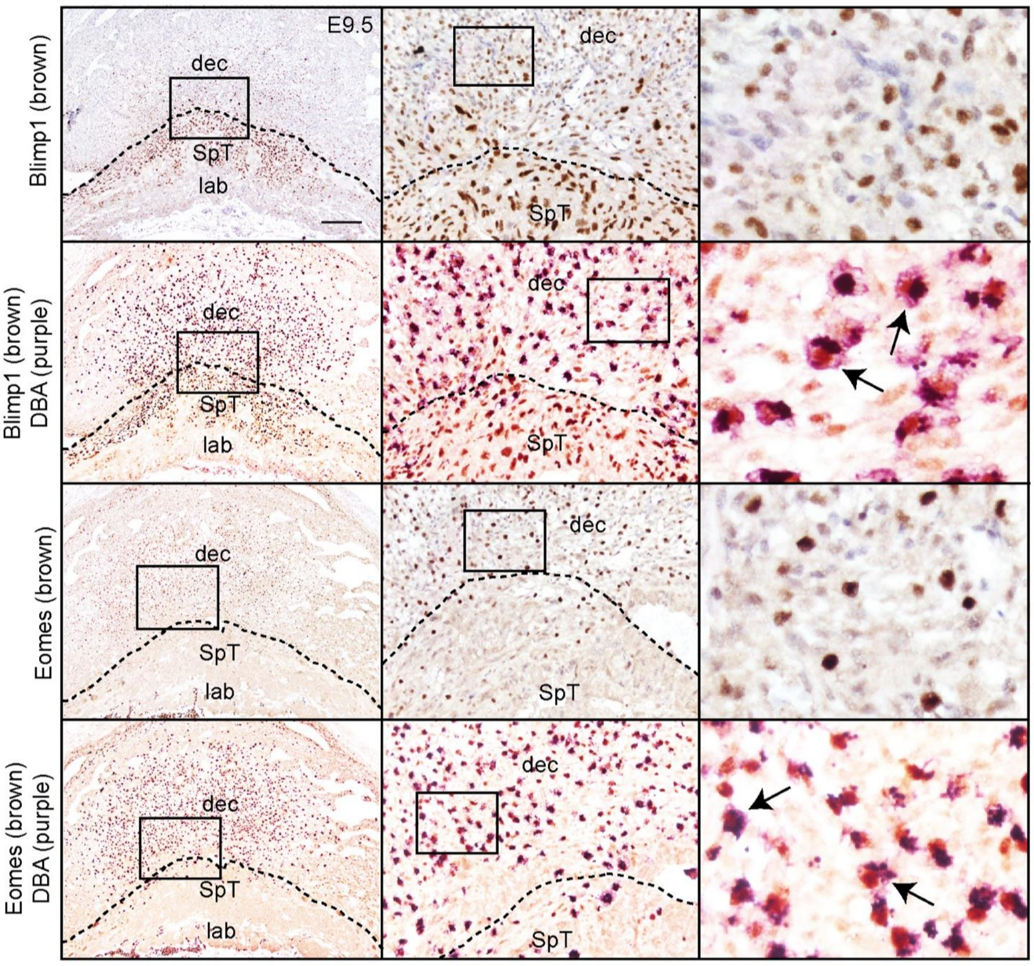

b

Rank order gene expression in uNK vs Spleen NK1.1+

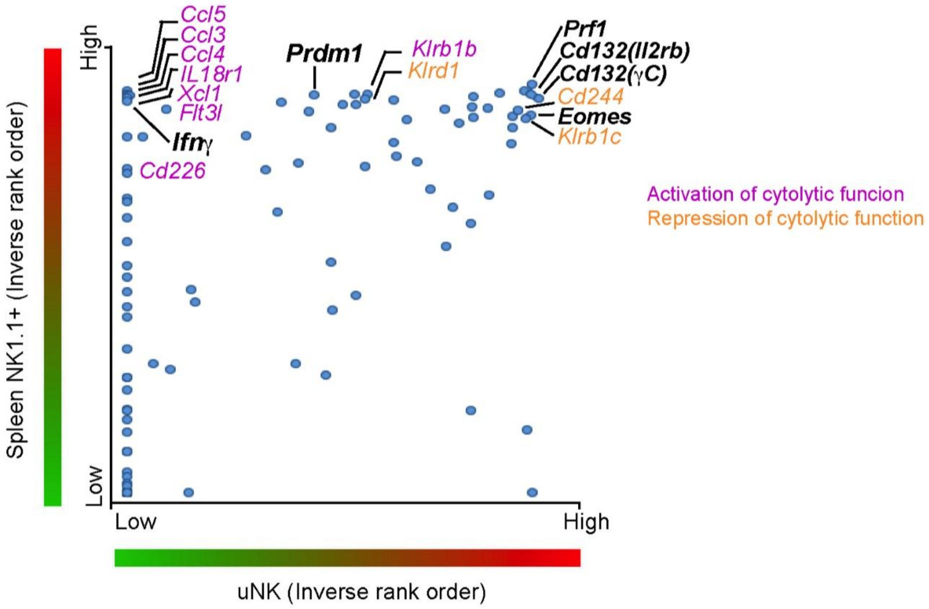

Supplementary Figure 2. uNK cells express *Prdm1* and other NK markers but lack expression of genes associated with activated cytolytic function.

(a) IHC for Blimp1 and Eomes with and without co-staining with uNK marker DBA lectin indicates that uNK cells express *Prdm1* and *Eomes*. Dotted lines demark embryonic (SpT layer) and maternal (decidua) tissues. Double stained cells are indicated with arrows. Abbreviates: dec - decidua; SpT - spongiotrophoblast; lab – labyrinth. Bar, 200 μm. (b) Comparison of NK cell related genes (based on GO annotation) rank ordered on median expression level from our uNK cell scRNA-seq vs. Spleen NK1.1+ cell RNA-seq (GEO accession GDS2957). Similar to spleen NK1.1+ cells, uNK cells express high levels of *Prf1*, *Cd122*, *Cd123* and *Eomes*, and moderate levels of *Prdm1*. In contrast, multiple genes associated with cytolytic function are only minimally expressed in the uNK cells.

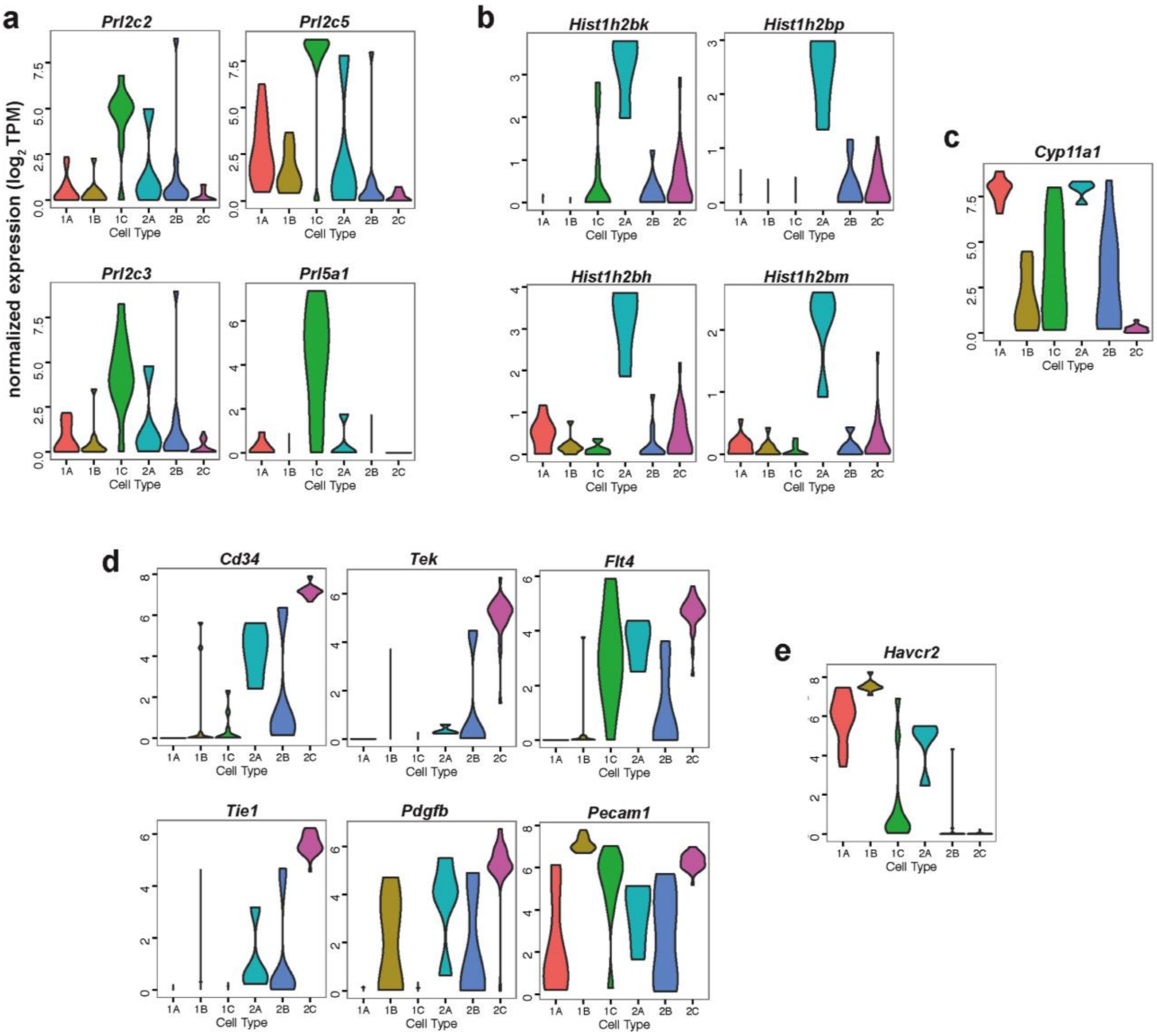

Supplementary Figure 3. Additional violin plots indicating: (a) *Prl* genes expressed in SpA-TGCs, but not GlyTs; (b) histone genes; (c) P-TGC marker *Cyp11a1*; (d) selected endothelial markers; (e) *Lgals9* receptor *Havcr2*.

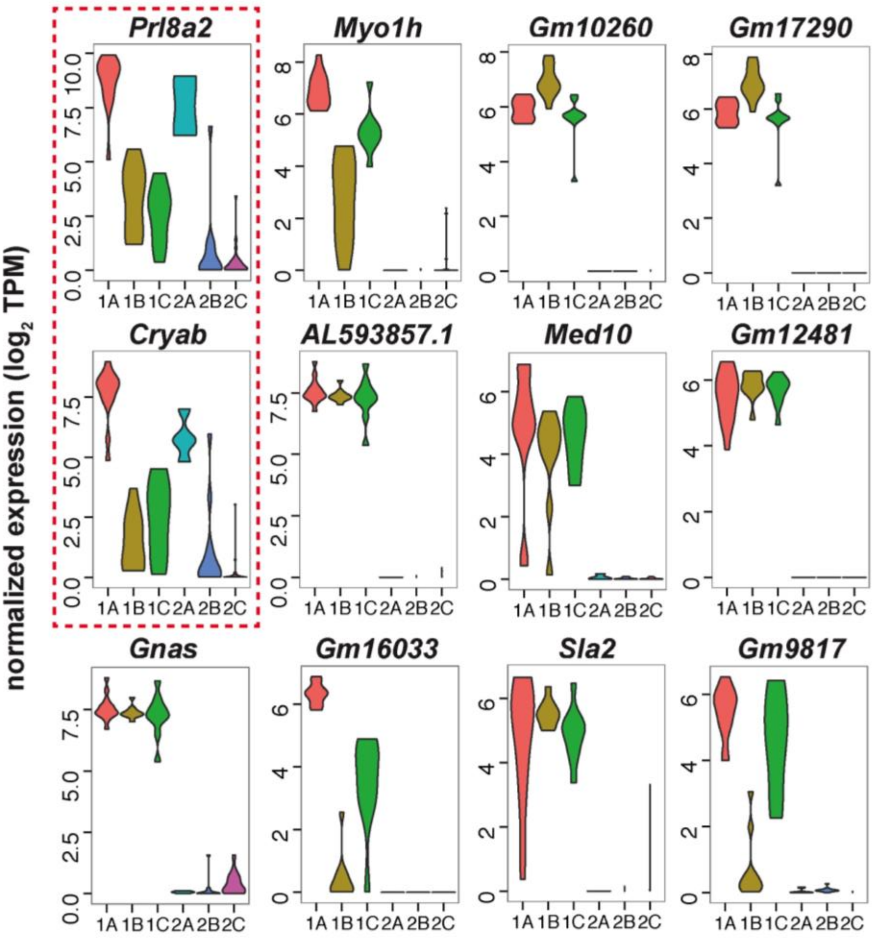

Supplementary Figure 4. Genes distinguishing decidual stromal cells (1A) and group 2A cells demonstrating that group 2A samples were not erroneous isolated as stromal:trophoblast doublets.

Shown are two commonly used decidual stromal markers which are expressed in 2A cells (red box) alongside a variety of genes which are highly expressed in decidual stromal cells (1A) but absent in 2A cells. 2A cells are therefore a distinct cell type genuinely expressing a subset of stromal markers.

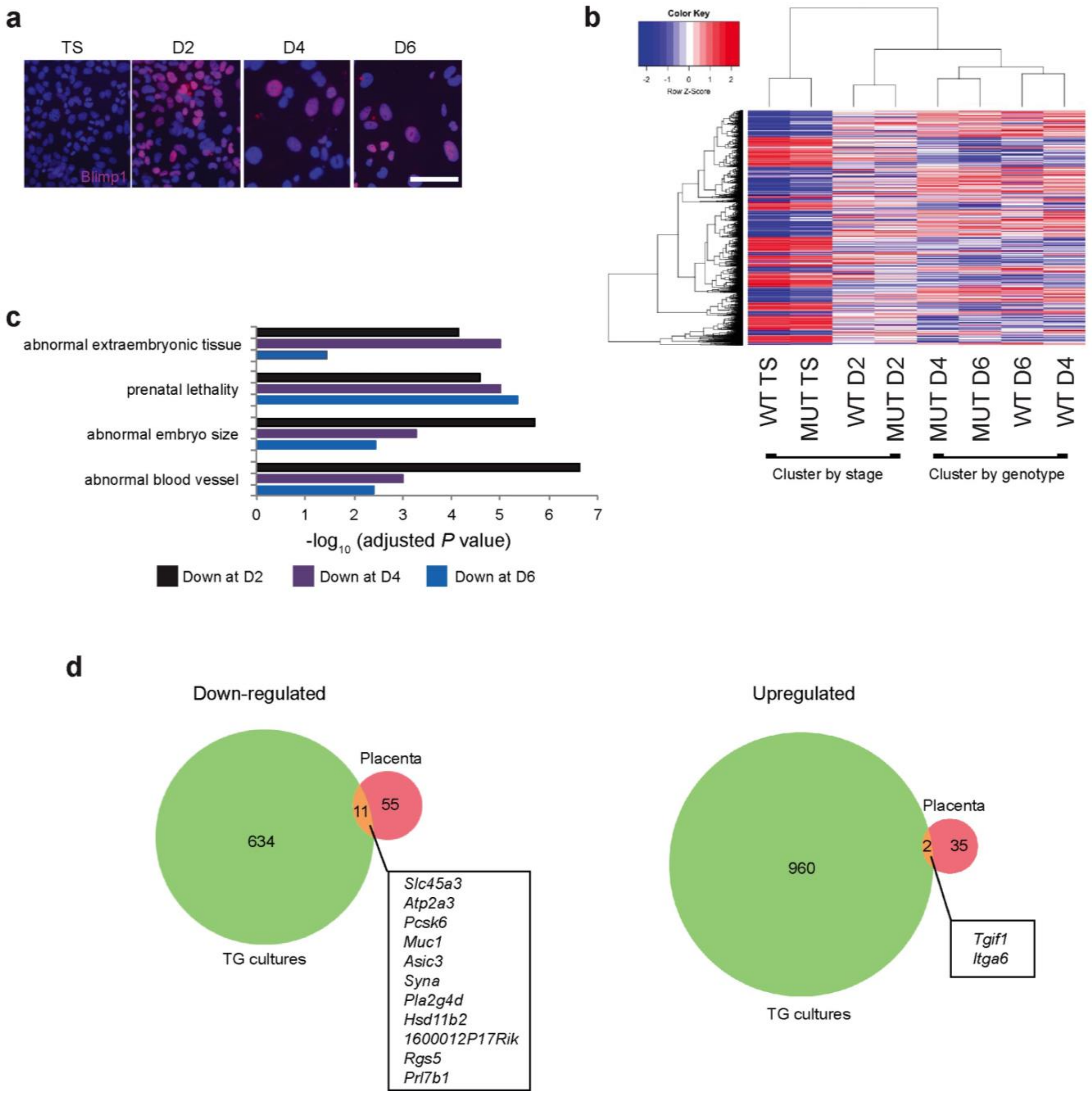

Supplementary Figure 5. Profiling Blimp1-dependent transcripts in differentiating TG cells.

(a) Immunoflourescence at two-day intervals during *in vitro* trophoblast differentiation corresponding to microarray timepoints reveals Blimp1 induced in diploid cells by day 2 and subsequently maintained in TGCs. Bar, 50 μm. (b) Hierarchical clustering of microarray profiles of mutant and wild type TS cell differentiation timecourse. (c) Functional annotation of genes downregulated ($P \leq 0.05$, Mann-Whitney U-test) at each timepoint. Reported $-\log_{10}$ adjusted P value computed from Fisher exact test by Enrichr. (d) Venn diagrams indicating

overlap between genes downregulated (left) or upregulated (right) in *Prdm1* mutant E9.5 placenta and differentiated TS cells at days 2, 4 and 6 collectively ($P \leq 0.05$, Mann-Whitney U-test).

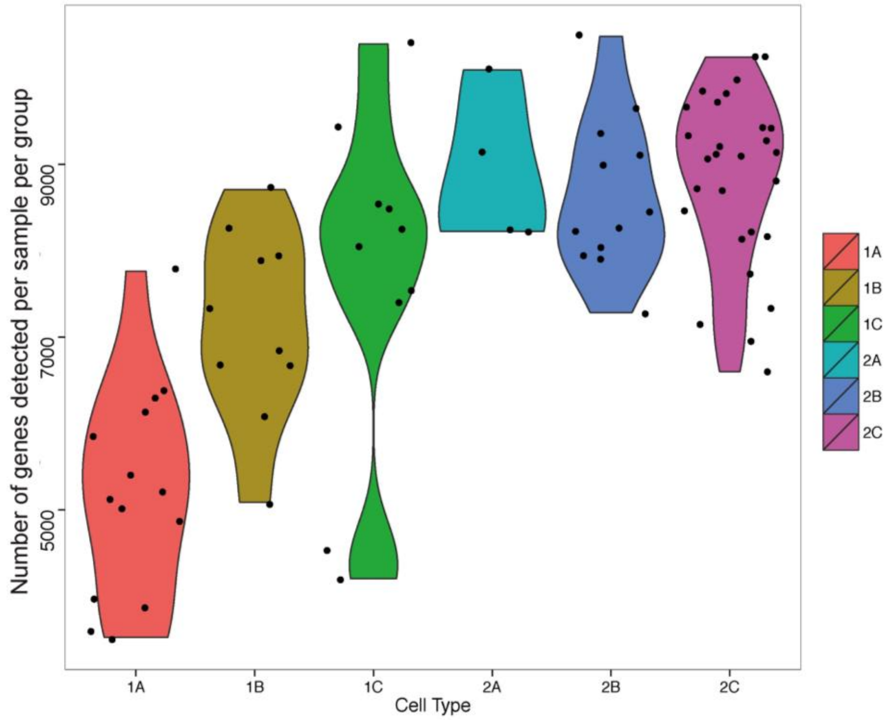

Supplementary Figure 6. Violin plot illustrating the number of genes detected per cell per group in our scRNA-seq data. 1A – Decidual stromal cells; 1B – uterine NK cells; 1C – SpA-TGCs; 2A – Novel *Prdm1+* TGCs; 2B – Progenitor trophoblasts; 2C – Foetal endothelial cells.

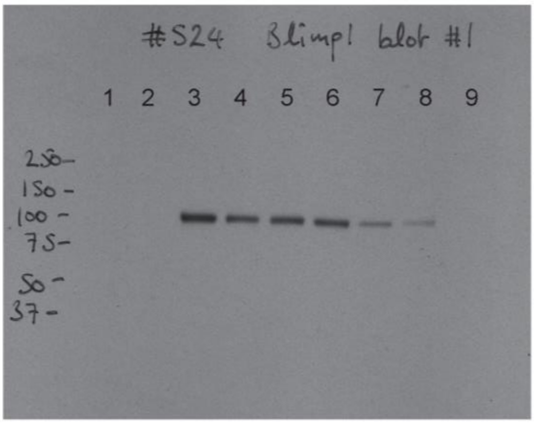

Lane 1 - Wild type TS cells
Lane 2 - Day 1 differentiation
Lane 3 - Day 2 differentiation
Lane 4 - Day 3 differentiation
Lane 5 - Day 4 differentiation
Lane 6 - Day 5 differentiation
Lane 7 - Day 6 differentiation
Lane 8 - E9.5 placenta
Lane 9 - STO mouse embryonic fibroblasts

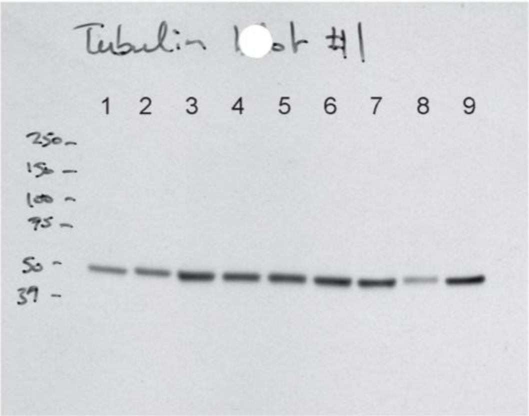

Supplementary Figure 7. Uncropped Western blots as shown in Figure 4a. Upper and lower blots probed with anti-Blimp1 and anti-Tubulin antibodies respectively**.**

Supplementary Table 1. Primers used in this study

Gene	Purpose	Forward primer	Reverse primer
Adm	Cloning	acatccggaTCCTCTTGGACTTTGGGGTTTTG	acgcgaatcgatACCTTTGGCTGGACAACAAG
Angpt4	Cloning	atcctaggatccAGCTTAACAGCCTCCAAG	acggcaatcgatCCGATGTTCCAGAATGTCA
Gapdh	qPCR	TGCACCACCAACTGCTTAGC	GGCATGGACTGTGGTCATGAG
Gjb3	qPCR	GGGGCTCTCCATCAGACATA	ACCTGCTAGCCACACTTGCT
Prdm1 #1	qPCR	TTGTGAGCCAAGCCATGTAA	GAGGTGCTCTGCTCTCTGGT
Prdm1 #2	qPCR	TTGATTCGGGTCAGATCCTC	AAGTGTGCCCAGTGTCACAA
Prdm1 #3	qPCR	GCCTCATTTATGCCCTGAGA	GGTGAATCAGGGTGCCTTTA
Prdm1 #4	qPCR	GGCTCCACTACCCTTATCCTG	TCCTTTTGGAGGGATTGGAGTC
Prdm1-Venus #1	qPCR	ACCCTGAAGCTGATCTGCAC	GGTCTTGTAGTTGCCGTCGT
Prdm1-Venus #2	qPCR	ACTCATCTCAGAAGAGGATCTG	CACAGTCGAGGCTGATCTCG
Prl7b1	qPCR	GTTAGCGAAATGCTCACCGAAG	AGTGGCAAAGTGTGGCAACTG
Tfap2c	qPCR	GTGAGGTCTTCTGCTCGGTC	CTCTTCTGAGCACACCTCCC

Supplementary Table 2. Summary of correctly paired mapped reads per samples.

Sample name	Properly paired reads	Sample name	Properly paired reads
Cell 1	34853538	Cell 40	42770792
Cell 2	45726070	Cell 41	29835451
Cell 3	43378758	Cell 42	43445714
Cell 4	34984103	Cell 43	35726464
Cell 5	36987994	Cell 44	28201660
Cell 6	42907706	Cell 45	28452007
Cell 7	42968631	Cell 46	37039781
Cell 8	39202283	Cell 47	54847668
Cell 9	35072620	Cell 48	62619164
Cell 10	55790064	Cell 49	68912278
Cell 11	47303801	Cell 50	70460732
Cell 12	48308072	Cell 51	55127464
Cell 13	45492107	Cell 52	35055694
Cell 14	50674157	Cell 53	34867804
Cell 15	49126995	Cell 54	43660870
Cell 16	50159315	Cell 55	37194051
Cell 17	66414161	Cell 56	47662674
Cell 18	47815616	Cell 57	37192782
Cell 19	42726266	Cell 58	45341817
Cell 20	47879845	Cell 59	33877782
Cell 21	52428475	Cell 60	63662152
Cell 22	47284164	Cell 61	72124638
Cell 23	55011128	Cluster 1	41559261
Cell 24	62978785	Cluster 2	49062841
Cell 25	46621289	Cluster 3	47626176
Cell 26	42615313	Cluster 4	49483438
Cell 27	42081646	Cluster 5	49974792
Cell 28	48407551	Cluster 6	69648934
Cell 29	26466855	Cluster 7	51807236
Cell 30	46546398	Cluster 8	50772598
Cell 31	30302627	Cluster 9	50831794
Cell 32	41104633	Cluster 10	44875659
Cell 33	32430706	Cluster 11	52402982
Cell 34	52411381	Cluster 12	45983800
Cell 35	42548963	Cluster 13	43007421
Cell 36	39351233	Cluster 14	55110218
Cell 37	43316459	Cluster 15	48859382
Cell 38	32306547	Cluster 16	52289117
Cell 39	37346367	Cluster 17	62186013
